# Effects of hTERT transfection on the telomere and telomerase of *Periplaneta americana* cells in vitro

**DOI:** 10.1186/s13568-023-01624-w

**Published:** 2023-10-21

**Authors:** Chenjing Ma, Xian Li, Weifeng Ding, Xin Zhang, Hang Chen, Ying Feng

**Affiliations:** 1 Key Laboratory of Breeding and Utilization of Resource Insects of National Forestry and Grassland Administration, Institute of Highland Forest Science, Chinese Academy of Forestry, Kunming, Yunnan Province 650224 China; 2 Nanjing Forestry University, Nanjing, Jiangsu Province 210037 China

**Keywords:** hTERT, Telomerase, Telomere, *Periplaneta americana*, Cell immortalization

## Abstract

Telomere and telomerase are crucial factors in cell division and chromosome stability. Telomerase activity in most cells depends on the transcription control by the telomerase reverse transcriptase (TERT). The introduction of an exogenous human TERT (hTERT) in cultured cells could enhance telomerase activity and elongate the lifespan of various cells. Telomere elongation mechanisms vary between insects and are complex and unusual. Whether the use of exogenous hTERT can immortalize primary insect cells remains to be investigated. In this study, we used a recombinant virus expressing hTERT to infect primary cultured cells of *Periplaneta americana* and evaluated its effects on insect cell immortalization. We found that hTERT was successfully expressed and promoted the growth of *P. americana* cells, shortening their doubling time. This was due to the ability of hTERT to increase the activity of telomerase in *P. americana* cells, thus prolonging the telomeres. Our study lays the foundation for understanding the mechanisms of telomere elongation in *P. americana*, and suggests that the introduction of hTERT into insect cells could be an efficient way to establish certain insect cell lines.

**Supplementary Information:**

The online version contains supplementary material available at 10.1186/s13568-023-01624-w.

## Key points

The recombinant *Autographa californica* multiple nucleopolyhedrovirus-human telomerase reverse transcriptase (AcMNPV-hTERT) gene was successfully expressed in *Periplaneta americana* cells.

The hTERT was successfully expressed in insect cells and promoted the growth of *Periplaneta americana* cells and shortened the doubling time of the cells.

The introduction of hTERT could increase telomerase activity and prolong the telomeres of *Periplaneta americana* cells.

**Supplementary Information:**

The online version contains supplementary material available at 10.1186/s13568-023-01624-w.

## Introduction

Insect cells have attracted great interest from the medical field as they offer high-level protein expression and can be used to develop new therapeutics (Cox 2012; Dias et al. 2021; Drugmand et al. 2012; Zhang et al. 2012). However, the establishment of insect cell lines is challenging and time-consuming (Murhammer 2007). Many insect cells require long-term primary cultures and are generally unable to undergo early subculturing due to a limited number of cell divisions, likely caused by low telomerase activity and short telomeres that rapidly drive them toward the restrictions of the ‘Hayflick limit’ (Hayflick and Moorhead 1961). Therefore, conventional methods of tissue cell culture have been used with limited success rates.

The telomerase is a ribonucleoprotein complex that possesses the capacity to synthesize telomeric DNA, thereby maintaining telomere length and facilitating indefinite cell division (Blasco 2005; Forsyth et al. 2002; Hathcock et al. 2005; Hiyama and Hiyama 2007). It plays an important role in maintaining cell viability and self-renewal (Allsopp et al. 2003). The telomerase complex consists of four essential components: telomerase reverse transcriptase (TERT), telomerase-associated protein (TEP), telomerase RNA (TR), and the catalytic subunit of telomerase (Revy et al. 2023). TERT is crucial for activating telomerase, a unique enzyme that differs from other reverse transcriptase in its ability to realign templates and continue synthesizing multiple DNA repeats (Farooqi et al. 2018; Kogan et al. 2006). Detecting telomerase activity in normal cells is difficult; however, a majority of the tumor and immortal cell lines exhibit telomerase expression, indicating a crucial role of telomerase in cancer initiation and cellular immortality (Bodnar et al. 1998; Zong et al. 2021). Telomerase activity is typically absent in mature somatic cells of vertebrates, whereas it has been demonstrated to be specifically expressed in immortal, cancerous, and germ cells. In these cell types, telomerase compensates for the shortening of telomeres during DNA replication and thereby stabilizes their length (Akincilar et al. 2016; Greider 2012). Contrarily, telomerase activity has been reported in invertebrate cells. Notably, it has been detected in Isoptera, Lepidoptera, Orthoptera, Hymenoptera, Tricoptera, and Coleoptera, and the TERT gene has been cloned (Gong et al. 2015; Korandova et al. 2014; Robertson and Gordon 2006; Sasaki and Fujiwara 2000). Studies have shown that the TERT gene is expressed in various instars and tissues during the growth and development of some insects (Zhang et al. 2020). However, the expression of the TERT gene does not improve the telomerase activity nor the ability of division of these insect cells, making growth and immortalization of insect cells in vitro very difficult. In our previous study, we confirmed this phenomenon, as tentatives to culture cells from the embryonic American cockroach *Periplaneta americana*, which contains high telomerase activity (Zhang et al. 2018), led to early senescence of the cells. This observation led to the hypothesis that TERT activity in insect cells may not be involved in the cell proliferation and immortalization processes in vitro.

Stable overexpression of TERT has been established in various cell cultures, including human mammary epithelial cells, mouse embryonic stem cells, and mouse embryonic fibroblasts (Armstrong et al. 2005; Geserick et al. 2006; Smith et al. 2003). The human TERT (hTERT) has been identified as a catalytic enzyme involved in telomere elongation (Masutomi et al. 2000). Thus, the introduction of the hTERT gene into primary cultured cells has become a method for overcoming cell culture difficulties, prolonging cell life (Liu et al. 2005), and obtaining rare tissue cell lines, such as human bone marrow mesenchymal stem cell line (Luna et al. 2021), human mesenchymal stromal cells (Madonna et al. 2021), human epithelial cells of renal proximal tubules (Wieser et al. 2008), and dairy goat mammary epithelial cell line (Shi et al. 2014). The hTERT immortalized cells have been commercialized because they present improved proliferation capacities. For example, the immortalized cell line hTERT-RPE1 launched by Clontech and Geron in the United States proliferates faster than normal cells, with an average of 5–6 PDs per week, and the total number of passages exceeds 150 generations. Three of these clones are more than 300 generations (Bian et al. 2011; Sakai et al. 2004). However, little research has been reported on the transfection of hTERT into insect cells. Furthermore, insects and humans are two completely different species. Whether the human TERT gene can be expressed in insects and play a role in increasing telomerase activity remains a subject for further investigation. Previous studies have demonstrated that introducing the hTERT gene into cultured cells led to their immortalization. Therefore, we hypothesized that hTERT expression into insect cells could help overcome the difficulty of proliferating and immortalizing these cells.

Telomerase activity is difficult to detect in the model insects *Bombyx mori* and *Drosophila melanogaster* to the lack of TERT translation in *Bombyx mori* and the absence of telomeric (TTAGG)n repeat structure in *Drosophila melanogaster* (Korandova et al. 2014; Sasaki and Fujiwara 2000). As such, these two model insects are not suitable for insect telomere research. Conversely, the telomere repeat sequence and telomerase structure of *Periplaneta americana* are consistent with those of most insects, possessing a (TTAGG)n repeat structure, and the telomere elongation mechanism involves a telomerase-dependent mechanism (TA) (Korandova et al. 2014). Additionally, our preliminary studies have demonstrated that telomerase activity is detectable in *P. americana* cells in vitro (Zhang et al. 2018), making *P. americana* cells a suitable surrogate for studying telomeres and telomerase in most insect cells.

In our previous study, we aimed at establishing an embryonic cell line from the American cockroach *P. americana* (Blattaria: Blattidae) (Zhang et al. 2018). The establishment of this cell line was time-consuming, taking over 3 years, and the cells frequently stopped proliferating and exhibited a stagnation period. Hence, in this study, a recombinant baculovirus carrying hTERT had been established with the Bac-to-Bac baculovirus expression system. Primary cultured cells from embryonic American cockroach were infected with recombinant baculovirus, and the growth characteristics, recombinant protein expression, and telomerase activity were compared with *P. americana* cells in vivo. This allowed us to evaluate the characteristics and mechanism of the hTERT action in insect cell immortalization: our results show that the expression of hTERT increases the capacity of *P. americana* cells to become immortalized in vitro.

## Materials and methods

### Virus

The *hTERT* gene (GenBank: NM_198253) was synthesized by Sangon Biotech (Sangon Biotech, Shanghai, China). The recombinant *Autographa californica* multiple nucleopolyhedrovirus- hTERT (AcMNPV-hTERT) was generated via the Bac-to-Bac baculovirus expression system (Life Technologies, Carlsbad, CA, USA; No. 10,359,016) obtained from Sangon Biotech. The P2 generation virus stock solution was utilized for testing purposes. The cells were infected with varying multiplicities of infection (MOIs) including 1, 5, and 10 infections.

### Cell line development and virus infection

An embryonic RIRI-PA1 cell line was developed as previously described from the embryonic tissue of *P. americana* (Zhang et al. 2018). After primary embryonic cultured cells had adhered to the culture flask (50 days from initiation) (RIRI-PA1-3), they were inoculated with the recombinant baculovirus AcMNPV-hTERT at MOIs of 1, 5, and 10 (RIRI-PA1-3 MOI 1, RIRI-PA1-3 MOI 5, RIRI-PA1-3 MOI 10), with a control group (RIRI-PA1-3 Control), treated with medium alone thus not inoculated, used as a negative control. Same inoculation was performed on RIRI-PA1 cells at 50 passages (RIRI-PA1-50) (Zhang et al. 2018) embryonic cells (RIRI-PA1-50 MOI 1, RIRI-PA1-50 MOI 5, RIRI-PA1-50 MOI 10, RIRI-PA1-50 Control).

### Cell growth characteristics

Upon entering the exponential growth phase, RIRI-PA1-3 and RIRI-PA1-50 cells were diluted 2 × 10^5^ cells/mL and subsequently seeded into 96-well plates. After all the cells had adhered, the AcMNPV-hTERT baculovirus was inoculated. Wells containing seeded cells were divided into four groups: three groups were inoculated with AcMNPV-hTERT at adjusted MOIs of 1, 5, and 10 respectively, and one group was incubated with fresh medium to serve as a negative control. After 6-hour inoculation of AcMNPV-hTERT, the medium containing the virus was replaced with fresh medium. The cells were cultured under constant temperature conditions of 28 °C in the absence of light. Seven and 14 days after infection, cytopathic effects of the inoculation were examined using a microscope (Olympus Corporation, Tokyo, Japan). Cell viability was detected using Cell Counting Kit-8 (CCK8) (MedChemExpress LLC., Shanghai, China). The protocol was executed in accordance with the manufacturer’s instructions. Briefly, CCK8 reagent was added to RIRI-PA1 cells and incubated for 3 h at 28 °C. The absorbance was measured at 450 nm using the microplate reader (Thermo Fisher Scientific Inc., Waltham, MA, USA). A standard curve was created for the different RIRI-PA1 cell numbers and absorbances. One and 10 days after infection, the RIRI-PA1 cell number was calculated using the standard curve.

The growth rate of RIRI-PA1 cells was evaluated from days 1–10 after viral infection using a previously established cell density analysis protocol (Murhammer 2007). The cells were inoculated into T-12.5 (12.5 cm^2^) flasks at a density of approximately 10^6^ cells/mL in a 5-mL medium. A suspension of cells was collected daily from three culture flasks and cell density was measured using a Vi-CELL cell viability analyzer (Beckman Coulter, Inc., Brea, CA, USA) every 24 h. A growth curve was established based on the average cell densities, and the cell population-doubling time (PDT) was calculated using Hayflick’s formula (Hayflick 1973). After the virus-infected primary cultured cells were maintained for 10 passages, the cell growth rate and PDT were tested again, and the cultured cells not infected with the virus were used as a control to detect the effect of the recombinant virus on the primary culture.

### Verification of the cell line origin

The Genomic DNA Isolation Kit (TransGen Biotech, Beijing, China) was utilized to extract total DNA from RIRI-PA1-3 and RIRI-PA1-50 cells (control and 7-day AcMNPV-hTERT MOI 10 infection groups). The mitochondrial cytochrome c oxidase subunit I gene (COI) was amplified via PCR (Bio-Rad Laboratories, Inc., Hercules, CA, USA) using EasyTaq® DNA Polymerase (TransGen Biotech) and the following primers: HCO, 5′-TAAACTTCAGGGTGACCAAAAAATCA-3′; and LCO, 5′-GGTCAACAAATCATAAAGATATTGG-3′. The PCR conditions were as follows: initial denaturation at 94 °C for 5 min; followed by 35 cycles of denaturation at 94 °C for 30 s, annealing at 54 °C for 30 s, and extension at 72 °C for 1 min; and a final extension step at 72 °C for 7 min. The amplified COI gene was identified through MEGA 4.1 software following sequencing by Sangon Biotech of the PCR products (Tamura et al. 2007).

### Quantitative real-time polymerase chain reaction (qPCR) analysis

After 7 and 14 days of infection with AcMNPV-hTERT, the total RNA of RIRI-PA1-3 and RIRI-PA1-50 cells was isolated using TRIzol reagent (Invitrogen, Life Technologies Corporation, Carlsbad, CA, USA). The RNA was reverse-transcribed into cDNA using the PrimeScript™ RT reagent kit (Takara Bio Inc., Shiga, Japan) in accordance with the manufacturer’s instructions. The primers were as follows: hTERT primer (hTERT-F: 5′-TATCGGAATCAGGCAGCACC-3′, and hTERT-R: 5′-TGGTCTGGCCTCTCTATGCT-3′), *P. americana* actin primer (actin-F: 5′-TAGTGCCTGGTTCTGTGGTG-3′, and actin-R: 5′-CAGCAACTTCTTCGTCGCAC-3′). Quantitative PCR (qPCR) was conducted using the PowerUp™ SYBR Green Master Mix (Applied Biosystems, Life Technologies Corporation), and analyses were performed on the Applied Biosystems QuantStudio 3 Real-Time PCR System (Applied Biosystem). The relative quantification of *hTERT* genes was determined by utilizing the 2^−ΔΔCt^ method with *actin* serving as an internal standard.

### Western blotting analysis

After 7 and 14 days of infection with AcMNPV-hTERT, the cellular proteins were extracted using WB cell lysis buffer (Beyotime Biotechnology, Shanghai, China) in accordance with the manufacturer’s instructions. Next, the Protein Quantitative Kit (Beijing ComWin Biotech Co., Ltd., Beijing, China) was used to determine the protein concentration of cell samples. Forty micrograms of protein were separated by sodium sulfate–polyacrylamide gel electrophoresis (10% acrylamide). The protein was transferred from the gel onto a polyvinylidene difluoride membrane (Merck Millipore Ltd., Tullagreen, Carrigtwohill, Co. Cork, Ireland) using the Mini Trans-Blot Module (Bio-Rad Laboratories). The membrane was probed with monoclonal Anti-FLAG® M2 Mouse Antibody (1:1000; Sigma-Aldrich, St. Louis, MO, USA) as primary antibody and incubated with Goat Anti-Mouse IgG (H + L) secondary antibody (1:2500; TransGen Biotech). Subsequently, the bands’ images were analyzed using ChemiDoc XRS+ (Bio-Rad Laboratories) after visualizing using the BeyoECL Plus enhanced chemiluminescence (Beyotime Biotechnology).

### Telomeric repeat amplification protocol (TRAP) assays

TRAP was employed to identify the telomeric repeat (TTAGG)n of insects using the following primers: TS primer: 5′-AAGCCATCGAGCAGAGTT-3′; Bm-CX: 5′-GTGTAACCTAACCTAACC-3′ (Sasaki and Fujiwara 2000). Specifically, we extracted cell protein and determined the protein concentration as described above. Incubated 10 mg of protein, TS primer, and dNTP at 30 °C for 60 min followed by heating at 95 °C for 5 min. The resulting products were subsequently mixed with dNTP, TS primer, Bm-CX primer, and Taq DNA polymerase (TransGen Biotech, Beijing, China) and amplified by PCR using a thermal cycler (Bio-Rad Laboratories) with 35 cycles at 94 °C for 30 s and 60 °C for 30 s. PCR products were electrophoresed on 12% non-denaturing polyacrylamide gels at 200 V for 45 min, followed by visualization of the signals using GelRed nucleic acid dyes (Sangon Biotech Shanghai). The corresponding signals were detected by analyzing digital images with ChemiDocTM XRS+ (Bio-Rad Laboratories).

The TRAP products generated in the initial step were amplified using TS and Bm-CX primers, along with PowerUp™ SYBR Green Master Mix (Applied Biosystems) and subjected to qPCR analysis on the Applied Biosystems QuantStudio 3 Real-Time PCR System (Applied Biosystems). The relative quantification of genes was determined utilizing the 2^−ΔΔCt^ method with actin as an internal standard.

### Cloning and sequencing of the TRAP products

The TRAP products were cloned and sequenced by Sangon Biotech. Briefly, the purified TRAP products were obtained using SanPrep Column DNA Gel Extraction Kit (Sangon Biotech) and subsequently subjected to cloning with Hieff CloneTM Zero TOPO-TA Cloning Kit (Shanghai YISHENG Biological Technology Co., Ltd., Shanghai, China). Among the positive colonies identified through colony hybridization, a random selection of 10 clones was subjected to sequencing using an ABI 3730 sequencer (Thermo Fisher).

### Quantitative fluorescence in situ hybridization (Q-FISH)

(TTAGG)_5_ probe fabrication and Q-FISH experiments were commissioned by Beijing FUTURE Biotech Co., Ltd., to conduct the test. The fluorescence intensity of the telomeres in cells was examined under a confocal laser scanning microscope (Fv10i; Olympus Corporation) (45 images, 45 cells/group). The fluorescence intensity was expressed in terms of normalized values of corrected total cell fluorescence (Rabanal-Ruiz et al. 2021).

### Statistical analyses

Statistical analyses were conducted using the GraphPad Prism software (version 9.0; Dotmatics, Boston, MA, USA). All values are presented as mean ± standard error of the mean (SEM). Student’s *t*-test was used for comparison between two groups and one-way analysis of variance was used for comparison among three or more groups. *P*-values of < 0.05 were considered statistically significant in all tests. All experiments were conducted in triplicate.

## Results

### hTERT expression in RIRI-PA1 cells promote growth

**Fig. 1:**
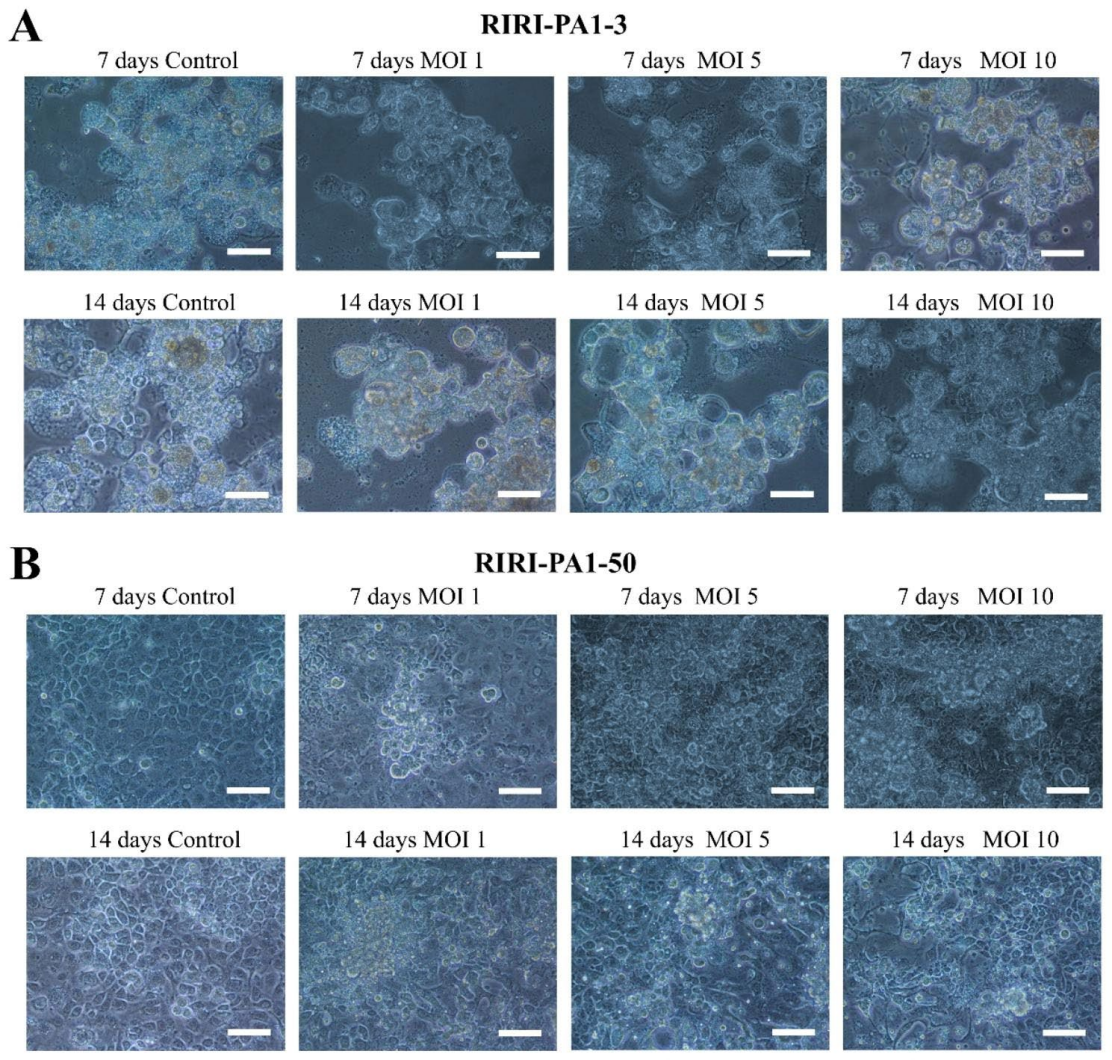
AcMNPV-hTERT infection in RIRI-PA1-3 and RIRI-PA1-50. **(A)** Infection of RIRI-PA1-3 cells with AcMNPV-hTERT for 7 and 14 days (200×) at MOIs of 1, 5, and 10. **(B)** Infection of RIRI-PA1-50 cells with AcMNPV-hTERT for 7 and 14 days (200×) at MOIs of 1, 5, and 10. Scale bar: 100 μm

**Fig. 2:**
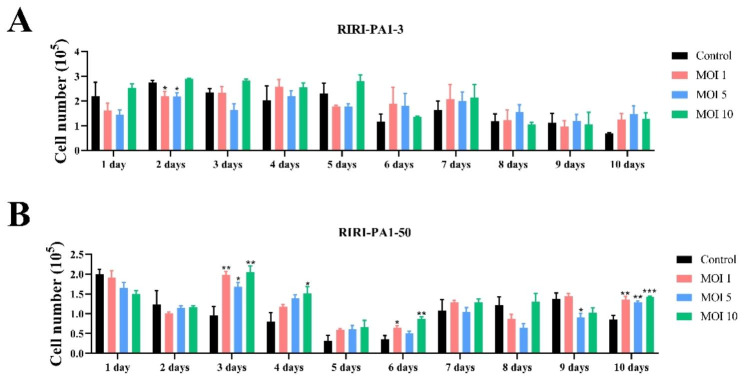
Changes in cell number in RIRI-PA1-3 and RIRI-PA1-50 after AcMNPV-hTERT infection for 1 to 10 days. **(A)** Changes in cell number in RIRI-PA1-3 cells with AcMNPV-hTERT infected for 1 to 10 days at MOIs of 1, 5, and 10. **(B)** Changes in cell number in RIRI-PA1-50 cells with AcMNPV-hTERT infected for 1 to 10 days at MOIs of 1, 5, and 10. Values are expressed as mean ± SEM. *P < 0.05, **P < 0.05, and ***P < 0.001, compared with control group

The state of growth and proliferation of the RIRI-PA1-3 and RIRI-PA1-50 inoculated with AcMNPV-hTERT indicated that expressing hTERT in *P. americana* in culture could accelerate their growth rate. The RIRI-PA1-3 and RIRI-PA1-50 cell lines exhibited sensitivity to AcMNPV-hTERT, resulting in typical cytopathic effects within the first 24 h post-infection; however, the cells quickly recovered and continued to grow and proliferate. The RIRI-PA1-3 and the RIRI-PA1-50 cells transfected with AcMNPV-hTERT at 7 and 14 days with different MOI values and expressing hTERT were more numerous and exhibited increased vitality after the cells had adapted to the virus compared to the cells without AcMNPV-hTERT infection (Fig. 1A and B). The transfected cells were translucent, and most cells adhered to the culture flasks. Compared with control cells not inoculated with hTERT, the number of RIRI-PA1-3 cells at the MOIs of 1, 5, and 10 exhibited an increasing trend at the 7–14 d of hTERT gene transfer (Fig. 2A).

The culture exhibited a lag phase of approximately 6 days following seeding, which corresponded to the cellular recovery and adaptation to the environment (Fig. 2A, Table S1). However, this was different for the RIRI-PA1-50 cells, where the lag time generally occurred on the first day after seeding, which may be due to a faster response of RIRI-PA1-50 cells to environmental changes (Fig. 2B, Table S1). After the initial lag time, the number of RIRI-PA1-3 cells gradually stabilized, and at MOI 10, this number has been consistently higher than the control group, which maintained a trend similar to what was observed for RIRI-PA1-50 cells. Combined with the growth state and growth curve of both cell types, we believed that MOI 10 had a better effect on cell growth promotion than MOI 1 and MOI 5.

### Establishment of the RIRI-PA-hTERT cell line

**Fig. 3:**
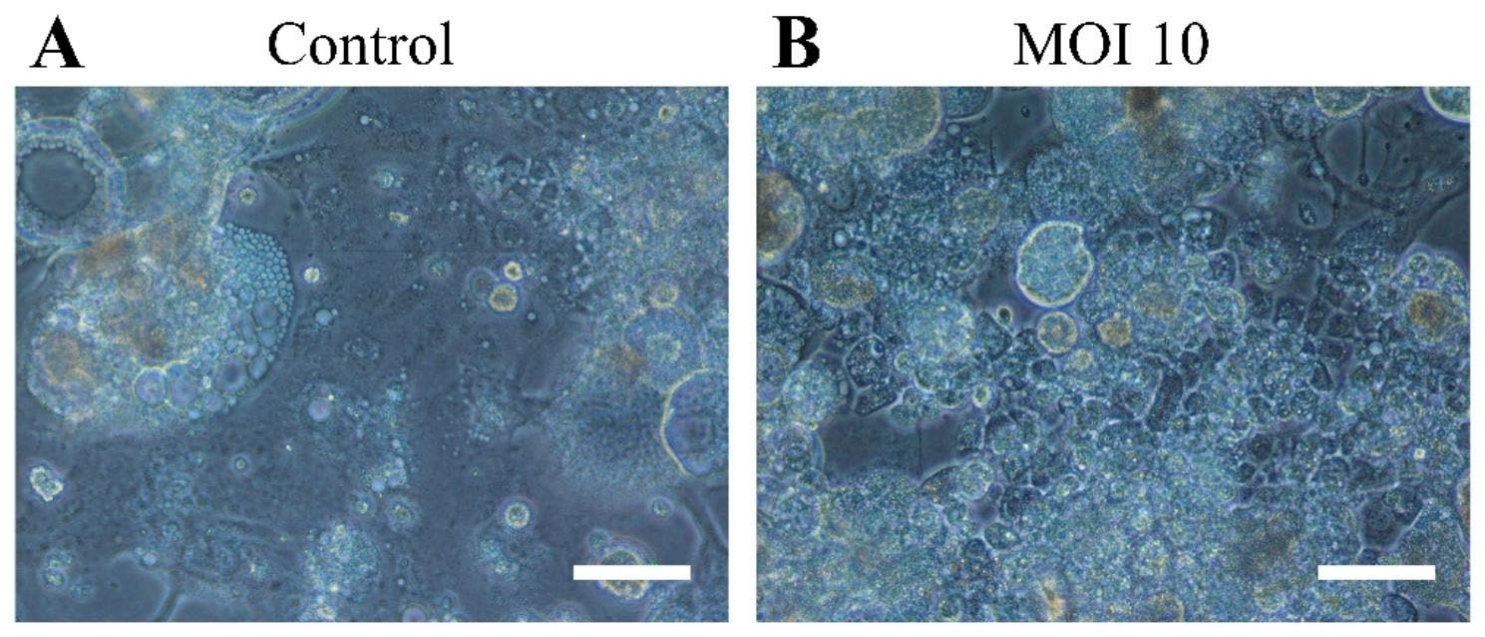
Cell growth state of RIRI-PA1-10 and RIRI-PA-hTERT-10. **(A)** Control group of RIRI-PA1-10 cells (200×). **(B)** Infection of RIRI-PA1-10 cells with AcMNPV-hTERT MOI 10 (200×). Scale bar: 100 μm

**Fig. 4:**
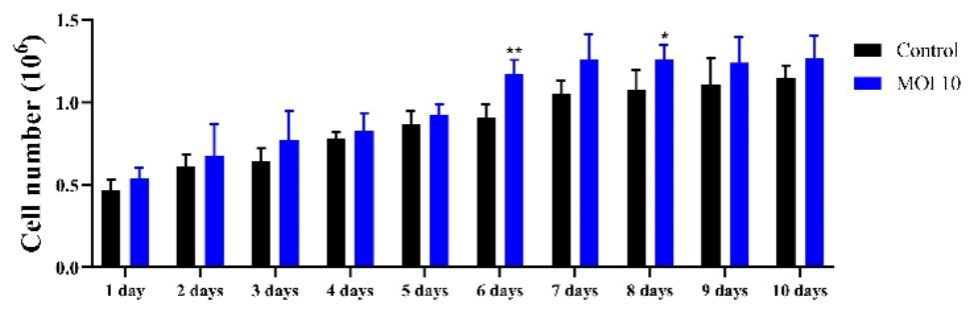
Growth curve of RIRI-PA1-10 and RIRI-PA-hTERT-10. Values are expressed as mean ± SEM. *P < 0.05 and **P < 0.05, compared with control group

Primary embryonic cells transfected with *hTERT* were passaged 40 times. The morphology of the primary cells transfected with hTERT was mostly oval, spindle-shaped or giant (Fig. 3A). Compared with control cells without AcMNPV-hTERT infection, RIRI-PA1-hTERT cells were numerous after transduction, forming growth clusters and presenting tight adherence to the flask, which are characteristics of a good cell growth status (Fig. 3B). In our previous study, we observed several lag phases during which the cells proliferated at a slower pace with most of the cells enable to go through the lag phases; the interval between each of the 10 first passages were ~ 20 days. RIRI-PA1 cells were cultured for ~ 40 passages over 3 years from the original culture (Zhang et al. 2018). In the present study, we found that hTERT can accelerate the growth of *P. americana* cells, causing most of the cells to go through the lag phases of growth. Therefore, our results indicated that the transduction of the *hTERT* gene can shorten the passage interval of *P. americana* cells. This effect was obvious in the first 10 passages, with an interval of ~ 12 days between each passage(MOI 10), and the doubling time of RIRI-PA1-10 shortened from 116.3 h without AcMNPV-hTERT infection to 74.6 h with AcMNPV-hTERT infection (MOI 10) (Fig. 4, Table S2) (RIRI-PA-hTERT-10 represents10th -passage of RIRI-PA1 embryonic cells with AcMNPV-hTERT infection). Hence, in the present study, RIRI-PA-hTERT were cultured for ~ 40 passages in ~ 1.8 years from the original culture.

### Verification of the cell line origin

**Fig. 5:**
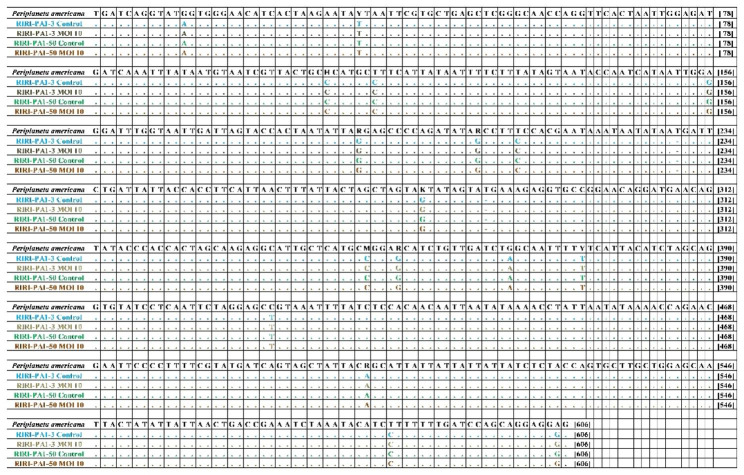
Alignment of the COI gene nucleotide sequences from *P. americana* embryos and RIRI-PA1 cells

To confirm that the properties of the primary cells transfected with hTERT were not altered after the transduction of hTERT, we performed cytochrome c oxidase subunit I (COI) gene sequencing on embryonic cells from *P. americana* and on the four lineages studied. Compared to *P. americana* embryos, COI gene sequences of RIRI-PA1-3 Control and RIRI-PA1-50 control cells (i.e., without AcMNPV-hTERT infection) presented a strong conservation rate of the sequences (Fig. 5), with 99.27% identity, confirming that our cell lines derive from *P. americana.* In addition, hTERT-expressing cells from lineages presented the same variations (Fig. 5), suggesting that hTERT expression does not alter gene sequences in these cells.

### Recombinant hTERT expression analysis

**Fig. 6:**
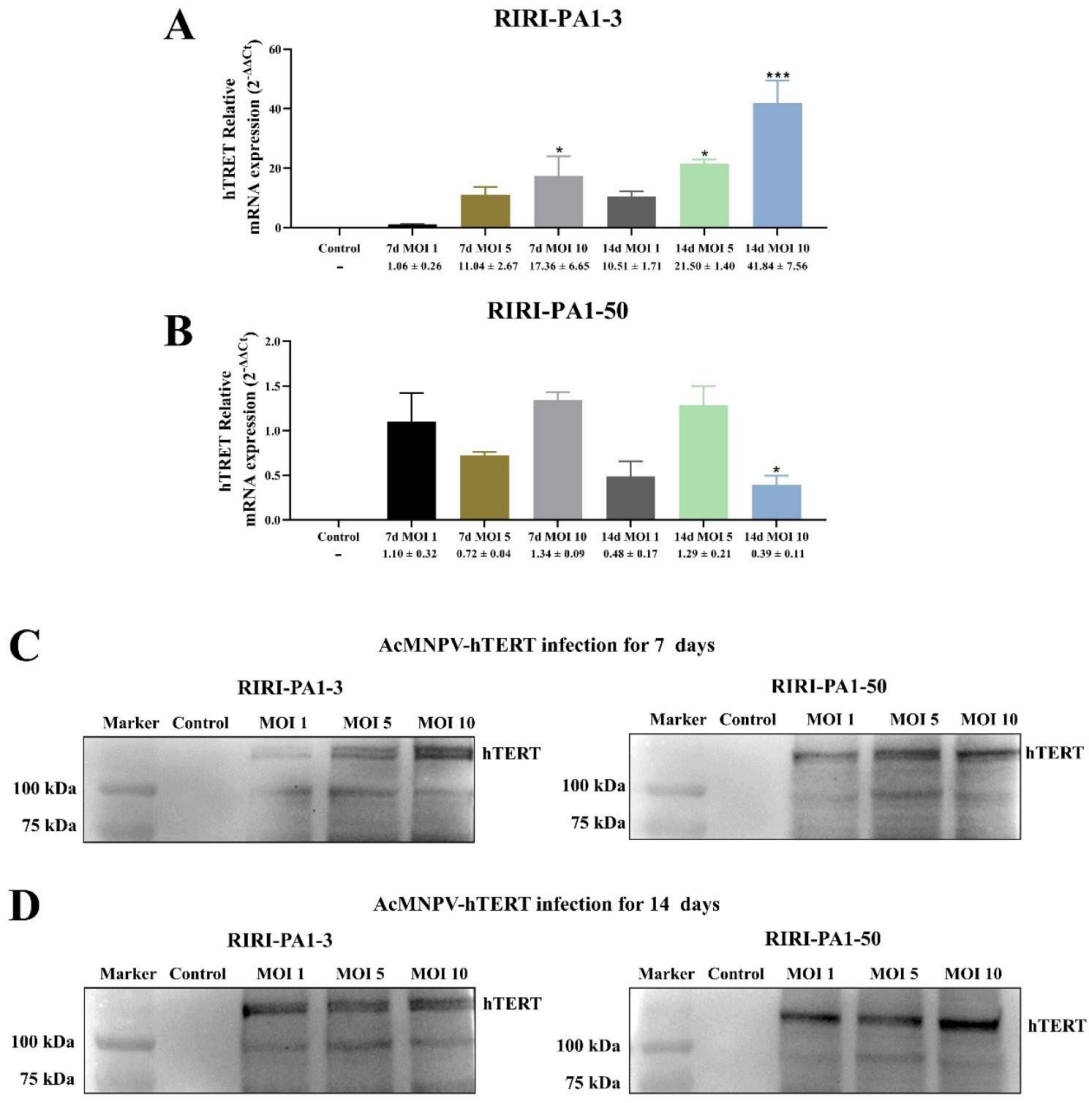
hTERT expression in RIRI-PA1-3 and RIRI-PA1-50 after AcMNPV-hTERT infection for 7 and 14 days. **(A–B)** hTERT relative mRNA expression in RIRI-PA1-3 (A) and RIRI-PA1-50 (B) infected with AcMNPV-hTERT for 7 and 14 days. Values are expressed as mean ± SEM. *P < 0.05 and ***P < 0.001, compared with 7 d MOI 1 group. **(C–D)** Western blot analysis of recombinant hTERT protein expression in RIRI-PA1-3 and RIRI-PA1-50 infected with AcMNPV-hTERT for 7 and 14 days

To assess any difference in the expression of hTERT depending on the cell line used and the MOI, we measured the level of hTERT mRNA in different MOI conditions and cell lines using qPCR seven and 14 days after transduction. We observed that, as expected, the hTERT relative mRNA expression in RIRI-PA1-3 cells was upregulated after both 7 and 14 days by compared with 7 d MOI 1 group, and this upregulation was MOI-dependent (*P < 0.05 and ***P < 0.001) (Fig. 6A, Table S3). However, the hTERT relative mRNA expression in RIRI-PA1-50 cells exhibited no significant upregulation change (Fig. 6B, Table S3). We subsequently performed western blot experiments to assess the expression of recombinant hTERT protein in RIRI-PA1 cells. Our findings demonstrate the successful expression of recombinant hTERT in both RIRI-PA1-3 and RIRI-PA1-50 cells, with a theoretical molecular weight of approximately 130.2 kDa, consistent with that of recombinant hTERT molecules. Notably, no band was detected in the negative control group that was not infected by AcMNPV-hTERT (Fig. 6C and D). After 7 and 14 days of infection, the hTERT expression was detected in RIRI-PA1-3 and RIRI-PA1-50 at MOI 1 to 10 (Fig. 6C and D).

### Detection of telomerase activity in RIRI-PA1 cells

**Fig. 7:**
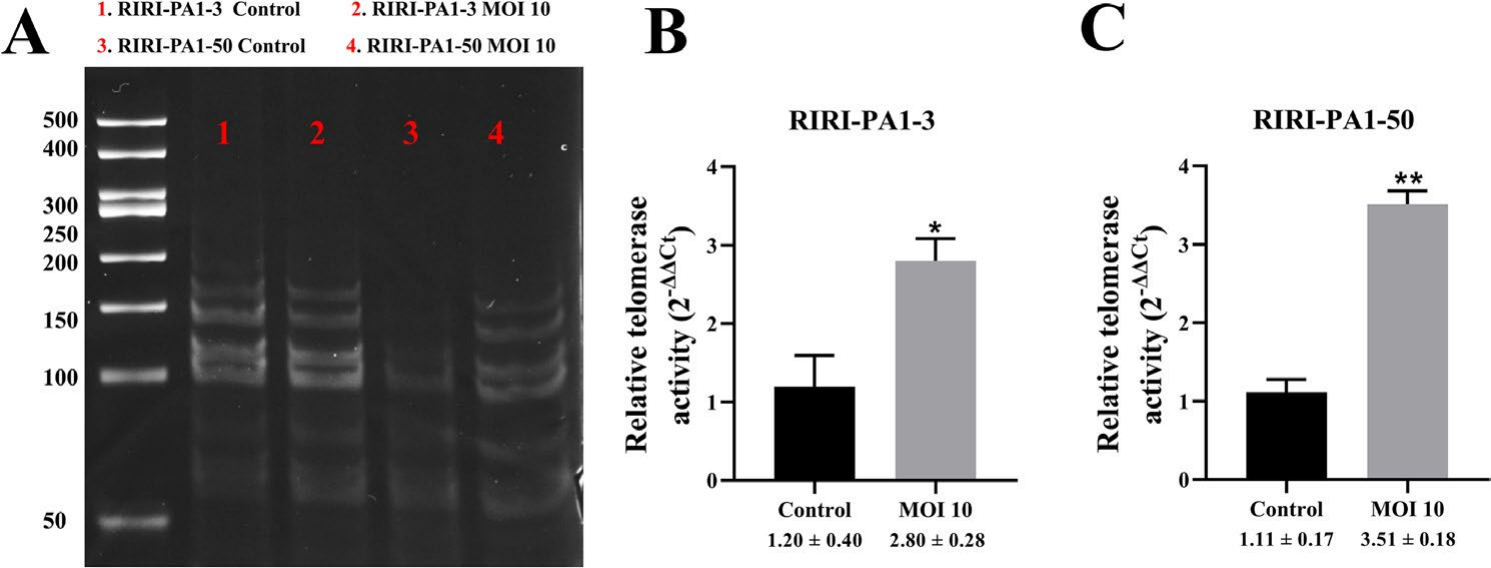
hTERT expression in RIRI-PA1-3 and RIRI-PA1-50 leads to an increased telomerase activity 7 days after infection. **(A)** Telomerase activity was detected using TRAP in RIRI-PA1-3 and RIRI-PA1-50 after AcMNPV-hTERT infection for 7 days. **(B)** Relative telomerase activity was detected using qPCR in RIRI-PA1-3 cells 7 days after AcMNPV-hTERT infection. Values are expressed as mean ± SEM, *P < 0.05, MOI 10 group compared with control group in RIRI-PA1-3 cells. **(C)** Relative telomerase activity was detected using qPCR in RIRI-PA1-50 cells 7 days after AcMNPV-hTERT infection. Values are expressed as mean ± SEM, **P < 0.01, MOI 10 group compared with control group in RIRI-PA1-50 cells

To test the telomerase activity in RIRI-PA1-3 and RIRI-PA1-50 infected for 7 days with AcMNPV-hTERT, we performed TRAP assays. Our results showed TRAP bands in all the four groups analyzed, which suggested that the RIRI-PA1 cell line has telomerase activity (Fig. 7A). Furthermore, qPCR analysis of telomerase activity revealed a significant increase in both RIRI-PA1-3 (control vs. MOI 10, *P < 0.05) (Fig. 7B) and RIRI-PA1-50 (control vs. MOI 10, **P < 0.01) after hTERT infection at MOI 10 (Fig. 7C), suggesting that hTERT expression in RIRI-PA1 cells increases telomerase activity.

### Telomere repeat sequence in RIRI-PA1 cells

**Fig. 8:**
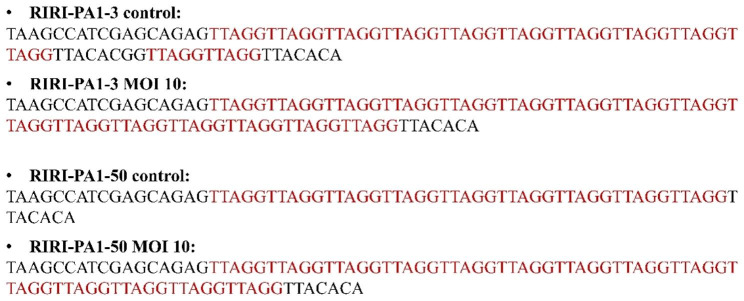
Sequences of TRAP products obtained upon AcMNPV-hTERT infection in RIRI-PA1-3 and RIRI-PA1-50 cells

After detection, the TRAP products from the previous experiment were cloned and sequenced. The resultant sequences of RIRI-PA1-3 and RIRI-PA1-50 cells 7 days after AcMNPV-hTERT infection were complete (TTAGG)n repeats (Fig. 8). The telomere of RIRI-PA1-3 and RIRI-PA1-50 was short, less than 100 bp (bp), whereas after AcMNPV-hTERT MOI 10 infection, the number of TTAGG repeats increased in RIRI-PA1-3 and RIRI-PA-50 cells, consistent with modification of the telomerase activity in RIRI-PA1 cells observed previously (Fig. 7B, C).

### Fluorescence detection of telomere repeats in RIRI-PA1 cells

**Fig. 9:**
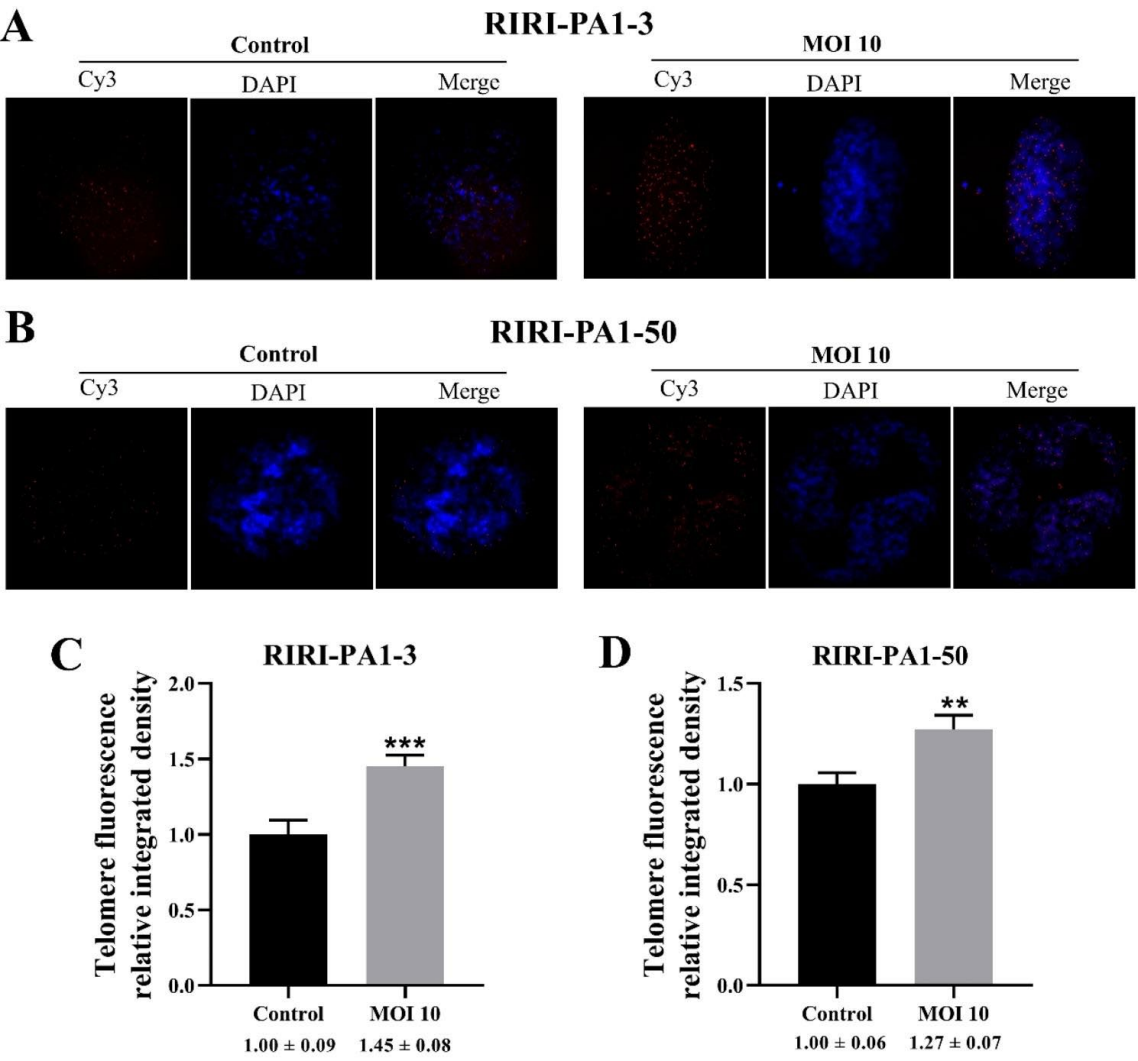
FISH mapping of RIRI-PA1-3 and RIRI-PA1-50 cells 7 days after AcMNPV-hTERT infection using the (TTAGG)5 telomeric probe. **(A)** Cy3-tagged telomeres in RIRI-PA1-3 cells 7 days after AcMNPV-hTERT infection. **(B)** Cy3-tagged telomeres in RIRI-PA1-50 cells 7 days after AcMNPV-hTERT infection. **(C)** Relative fluorescence-integrated densities of Cy3-tagged telomeres in RIRI-PA1-3. Values are expressed as mean ± SEM, ***P < 0.001, MOI 10 group compared with control group in RIRI-PA1-3. **(D)** Relative fluorescence-integrated densities of Cy3-tagged telomeres in RIRI-PA1-50. Values are expressed as mean ± SEM, **P < 0.01, MOI 10 group compared with control group in RIRI-PA1-50

Detection of telomere (TTAGG) repeats at the end of chromosome by Q-FISH analysis. The results revealed that all RIRI-PA1 cells were positively labeled in the telomeric regions, revealing the presence of the canonical insect sequence (TTAGG) (Fig. 9A and B). The degree of telomeres (fluorescence-integrated density of Cy3-tagged telomeres) in RIRI-PA1 cells was examined 7 days after AcMNPV-hTERT infection. The Cy3 fluorescence revealing the telomeres significantly increased in in both RIRI-PA1-3 cells (control vs. MOI 10, ***P < 0.001) and RIRI-PA1-50 cells (control vs. MOI 10, **P < 0.01) after hTERT infection at MOI 10 (Fig. 9C and D). This result confirmed that AcMNPV-hTERT infection increases the length of telomeres in hTERT-expressing RIRI-PA1 cells.

## Discussion

Telomeres are specialized structures composed of repetitive DNA sequences and associated proteins that cap the ends of linear chromosomes (Blackburn 1991). The (TTAGGG)n sequence is generally conserved across Metazoa, except for nematodes and arthropods, although insect telomere sequences can exhibit variations (Gomes et al. 2010). Indeed, while most insects have the (TTAGG)n telomere motif, such as Apidae, Formicidae, and American cockroach (Mohan et al. 2011; Sahara et al. 1999; Sasaki and Fujiwara 2000; Teixeira et al. 2022; Traut et al. 2007; Wurm et al. 2011), all dipteran and odonatan species lack the TTAGG repeat (Sahara et al. 1999), the chromosomal sequence being absent in numerous families of Hymenoptera (Menezes et al. 2017), and the Tingidae family presenting the (TTAGG)n motif but not the mechanism for maintaining telomere integrity (Golub et al. 2022). Moreover, some Coleoptera families like *Tribolium castaneum* and *Tenebrio molitor* constitutive of TCAGG repeats instead of TTAGG (Kuznetsova et al. 2019; Louis et al. 2014). Telomere structure varies in size, ranging from a mere 36 base pairs to several kilobases in length within the human genome (Greider 1996). In almost all animals and humans, telomere length shows a correlation with lifespan (Gomes et al. 2010), and telomere length reduction leads to cellular senescence or the arrest of cell division (D’Aiuto et al. 2003; de Lange 2005). Therefore, it is essential to compensate for the critical telomere shortening by adding telomeric repeats (Louis et al. 2014). But there are exceptions, such as the telomere length of *Lagomorph*, which is not related to its senescence or lifespan (Forsyth et al. 2005).

**Fig. 10:**
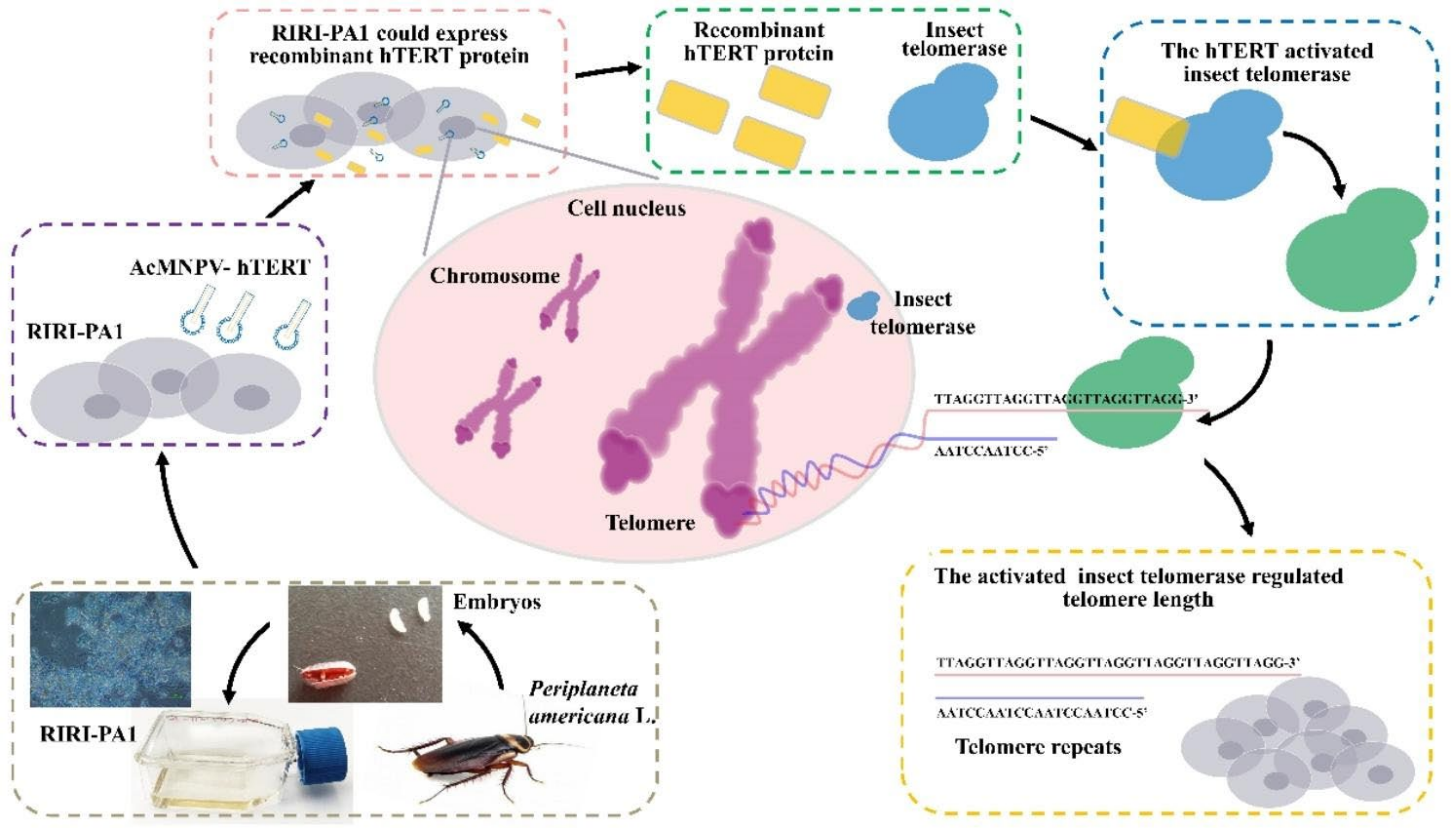
Flow-chart summary of the article

Telomere formation in insects varies and is unusual, suggesting that the telomere regions are maintained without telomerase activity (Sasaki and Fujiwara 2000). In different phylogenetic branches, the (TTAGG)n motif has undergone repeated loss and is likely to have been replaced by alternative mechanisms of telomere elongation (Frydrychova and Marec 2002). Some insects may have other, yet unknown, telomere organization (Golub et al. 2022). Therefore, it is worth investigating the role of the prolongation of telomeric nucleotide sequences in maintaining cell viability and increasing cell lifespan. In order to create an immortalized American cockroach’s cell line, we infected RIRI-PA1 cultured cells with a baculovirus that contained the human gene of the hTERT. We found that telomere prolongation in RIRI-PA1 cells is linked to cell lifespan. RIRI-PA1-3 and RIRI-PA1-50 cells presented complete but short (TTAGG)n repeats (< 100 bp). Using biochemical, biomolecular, and direct observations procedures, we observed that after 7 days of AcMNPV-hTERT MOI 10 infection, the cell number was increased compared to noninfected cells, as was the telomerase activity and TTAGG repeats number (Fig. 10). Altogether, our results indicate that the prolongation of cell lifespan could be related to telomere elongation regulated by telomerase activation.

There are two main mechanisms of telomere elongation in insects: one is the alternative lengthening of telomeres (ALT), characterized by telomerase-independent mechanisms of telomere maintenance and found in telomere- and telomerase-absent insects, such as *Diptera* (Biessmann and Mason 1997; Pardue and DeBaryshe 1999); the other relies on the activity of telomerase and retrotransposons (TA), which means that telomeres are maintained by telomerase. The latter remains predominant (Cimino-Reale et al. 2017), whereas ALT functions as a backup mechanism fulfilling a similar role. However, some insects possess distinct and often obscure telomeric sequences, implying the coexistence of telomerase-dependent mechanisms (TA) and alternative telomere lengthening mechanisms as backup for telomere maintenance (Kuznetsova et al. 2019). Studies have demonstrated that insects with (TTAGG)n motif telomeres mostly maintain telomere length by the TA mechanism (Cimino-Reale et al. 2017; Kuznetsova et al. 2019). In most eukaryotes, telomeres are maintained by telomerase, an enzyme possessing reverse transcriptase activity that facilitates chromosome end repair. TERT is a multifaceted and catalytically active constituent of the telomerase-associated protein machinery (Farooqi et al. 2018). In the process of cellular senescence, the activation of telomerase plays a key role, whereas the telomerase expression is mainly affected by TERT (Bodnar et al. 1998). In insects, the expression of TERT is not stable, and the poor transcription of the reverse transcriptase gene may account for the undetectable levels of its enzymatic activity (Osanai et al. 2006). In addition to low expression levels, the structure of insect TERT genes vary among species. The amino acid sequence of TERT displays limited conservation across distantly related species, except for a few catalytic sites (Louis et al. 2014). These features suggest that expressing TERT gene from insect to insect may not enhance the telomerase activity. Louis and Becker’s research confirmed this inference. They employed the baculovirus expression system to facilitate the expression of *TERT* gene from *Bombyx mori* and *Spodoptera frugiperda* in *B. mori* and Sf9 cells. They observed that telomerase activity was undetectable in these cells, indicating that the weak activity that resulted from low expression of the *TERT* gene within the organism. But what if the human TERT gene is expressed? Can the expression of this gene in insect cells increase their telomerase activity? The present study confirms that expressing hTERT in insect cells is feasible. The introduction of the hTERT gene into primary cultures has been proven to facilitate cell immortalization (Liu et al. 2005), although this has never been verified in insect cells. Furthermore, how would hTERT work in insect cells? We speculated that this should be related to the intracellular protein and gene structure of hTERT. Studies have indicated that the various proteins comprising the telomerase-associated machinery operate in a coordinated and orchestrated fashion to ensure proper maintenance of chromosomal telomere length (Lim and Cech 2021). The findings pointed to a pool of unassembled telomerase components (Farooqi et al. 2018). However, these components possess the capability to self-assemble into functional machinery in response to cellular demands. Our research indicated that the nucleotide sequence of the telomerase in *P americana* was changed after the introduction of the *hTERT* gene. Telomerase activity can be quantified in vitro through a primer extension assay, the TRAP assay, during which telomerase synthesizes telomeric repeats onto oligonucleotide primers (Kim et al. 1994). Telomerase adds telomeric repeats onto the nontelomeric oligonucleotide primer TS. The telomerase products are specifically amplified by PCR using the upstream primer TS and downstream primer CX (Kim et al. 1994). The TRAP of insects’ telomeric repeat (TTAGG)n detect procession was modified from mammals (Sasaki and Fujiwara 2000). Our results indicated that the RIRI-PA1 cell line had telomerase activity and that telomerase mRNA was upregulated in the AcMNPV-hTERT MOI 10 infection group, indicating that AcMNPV-hTERT MOI 10 infection increased telomerase activity in RIRI-PA1 cells. Then, we cloned the TRAP products and observed that AcMNPV-hTERT MOI 10 infection also increased the number of (TTAGG) repeats of RIRI-PA1 cells. The Q-FISH results had similar trends, and this suggests a relationship between hTERT introduction and telomere length regulation. These results indicate that there may be proteins and miRNAs as unassembled components present in insect cells that may have the capacity to work with hTERT. But we think the most important reason behind hTERT effectiveness in insect cells is that the terms of the structure of *TERT*, with the low conservation in insect *TERT* genes, and the differences between different species are large. The *TERT* gene in certain insect species, such as *B. mori* and *T. castaneum*, lacks introns but contains upstream ATG codons and no N-terminal GQ motifs (Osanai et al. 2006) and these insects all show a weak telomerase activity. But there are introns and N-terminal GQ motifs in hTERT, the structure of hTERT is more similar to the insect *TERT* genes which show telomerase activity, such as *Apis mellifera.* Furthermore, we introduced the *hTERT* gene into insect cells with its core promoter, which is essential for its transcription (Hafezi et al. 2021). This could explain why *hTERT* is expressed and functional in *P. americana* cells, but this inference needs to be verified in other insect cells.

*P. americana* (Blattidae: Blattaria) is one of the most widely distributed and common indoor sanitation pests in the world. It is also an important medicinal insect with antibacterial (Ali et al. 2017), antitumor (Zhao et al. 2017), and anti-inflammatory and analgesic (Nguyen et al. 2020) properties. It also promotes wound healing (Li et al. 2019), and improving immunity (Luo et al. 2014). The study of Sasaki and Fujiwara demonstrated that *P. americana* exhibits a telomerase activity that can be detected in somatic and germ cells at the same stage of its adult. It is inferred that the telomerase function of the adult stage is related to the telomerase activity. There is no tissue specificity of strength or weakness (Sasaki and Fujiwara 2000). A study of the telomerase activity of *P. americana* at different developmental stages showed that it gradually weakened with the development of the American cockroach and is closely related to tissue development (Korandova et al. 2014). By transfecting exogenous *hTERT* into in vitro culture of *P. americana*, we can enhance their telomerase activity, lengthen their telomeres, and increase their proliferation rate. Hence, it can be hypothesized that the activity of insect telomerase is closely associated with cellular proliferation. This study confirmed that the *hTERT* gene can promote the immortalization of insect cells already utilizing a telomerase lengthening telomeres mechanism of. Whether the same effect can be observed in insects that use other mechanisms to lengthen telomeres will require further investigation.

### Electronic supplementary material

Below is the link to the electronic supplementary material.

Supplementary Material 1

## Data Availability

The data that support the findings of this study are available in the Supplementary Materials of this article.
