# Supplementary material for: Effects of hTERT transfection on the telomere and telomerase of Periplaneta americana cells in vitro
Source: AMB Express. 2023 Oct 21;13:118. doi: 10.1186/s13568-023-01624-w (PMC10590340; doi:10.1186/s13568-023-01624-w)
Supplement: Supplementary file 1 — Supplementary Material 1 [file 13568_2023_1624_MOESM1_ESM.pdf]

Supplementary materials

Journal name

AMB Express

Manuscript Title

Effects of hTERT transfection on the telomere and telomerase of *Periplaneta americana* cells *in vitro*

Author names

Chenjing Ma[a], Xian Li[a], Weifeng Ding[a], Xin Zhang[a, *], Hang Chen[a], Ying Feng[a]

Affiliations

[a]Key Laboratory of Breeding and Utilization of Resource Insects of National Forestry and Grassland Administration, Institute of Highland Forest Science, Chinese Academy of Forestry, Kunming, Yunnan Province, 650224, China.

Chenjing Ma, E-mail: machenjing@caf.ac.cn

Xian Li, E-mail: lixian@caf.ac.cn

Weifeng Ding, E-mail: dingwf@caf.ac.cn

Xin Zhang, E-mail: zhangxin@caf.ac.cn

Hang Chen, E-mail: stuchen6481@gmail.com

Ying Feng, E-mail: rirify@139.com

Corresponding Author

*Xin Zhang, Associate Researcher, Institute of Highland Forest Science, Chinese Academy of Forestry, Kunming, Yunnan Province, China. E-mail: zhangxin@caf.ac.cn.

Present/permanent address

Institute of Highland Forest Science, Chinese Academy of Forestry, Longyuan Road, Panlong District, Kunming, Yunnan Province, 650224, China.

Table S1: The data of Fig. 2.

Table S2: The data of Fig. 4.

Table S3: The data of Fig. 6A and B.

Table S1. Changes in cell number in RIRI-PA1-3 and RIRI-PA1-50 after AcMNPV-hTERT infection for 1 to 10 days. Cell number (10^5)

Groups	1 day	2 days	3 days	4 days	5 days	6 days	7 days	8 days	9 days	10 days
						**RIRI-PA1-3 cells**				
**Control**	2.19 ± 0.57	2.74 ± 0.08	2.34 ± 0.17	2.02 ± 0.59	2.30 ± 0.41	1.16 ± 0.31	1.64 ± 0.35	1.18 ± 0.30	1.12 ± 0.38	0.68 ± 0.04
**MOI 1**	1.62 ± 0.28	2.19 ± 0.20*	2.32 ± 0.26	2.57 ± 0.30	1.77 ± 0.05	1.88 ± 0.67	2.07 ± 0.59	1.23 ± 0.41	0.97 ± 0.24	1.27 ± 0.23
**MOI 5**	1.44 ± 0.20	2.18 ± 0.16*	1.63 ± 0.26	2.18 ± 0.22	1.76 ± 0.12	1.81 ± 0.49	2.00 ± 0.37	1.55 ± 0.29	1.19 ± 0.26	1.46 ± 0.35
**MOI 10**	2.54 ± 0.15	2.90 ± 0.02	2.82 ± 0.07	2.55 ± 0.18	2.82 ± 0.25	1.36 ± 0.03	2.13 ± 0.53	1.05 ± 0.09	1.05 ± 0.49	1.27 ± 0.24
						**RIRI-PA1-50 cells**				
**Control**	1.99 ± 0.13	1.23 ± 0.36	0.95 ± 0.23	0.79 ± 0.23	0.32 ± 0.13	0.35 ± 0.09	1.08 ± 0.28	1.22 ± 0.20	1.37 ± 0.15	0.85 ± 0.10
**MOI 1**	1.91 ± 0.17	1.01 ± 0.04	1.98 ± 0.09**	1.17 ± 0.06	0.59 ± 0.02	0.65 ± 0.05*	1.29 ± 0.05	0.86 ± 0.12	1.44 ± 0.07	1.36 ± 0.08**
**MOI 5**	1.65 ± 0.14	1.14 ± 0.05	1.67 ± 0.11*	1.40 ± 0.08	0.61 ± 0.09	0.51 ± 0.05	1.05 ± 0.11	0.65 ± 0.10	0.90 ± 0.10*	1.29 ± 0.03**
**MOI 10**	1.50 ± 0.08	1.17 ± 0.03	2.05 ± 0.15**	1.51 ± 0.17*	0.66 ± 0.16	0.86 ± 0.06**	1.29 ± 0.08	1.30 ± 0.21	1.03 ± 0.11	1.42 ± 0.02***

Values are expressed as mean ± SEM. *P<0.05, **P<0.05, and ***P<0.001, compared with control group.

Table. S2. Growth curve of the hTERT-transfected of RIRI-PA1-10 cells. Cell number (10^6)

Groups	1 day	2 days	3 days	4 days	5 days	6 days	7 days	8 days	9 days	10 days
**Control**	0.47 ± 0.07	0.61 ± 0.07	0.64 ± 0.08	0.78 ± 0.04	0.87 ± 0.08	0.91 ± 0.08	1.05 ± 0.08	1.08 ± 0.12	1.11 ± 0.16	1.15 ± 0.08
**MOI 10**	0.54 ± 0.06	0.68 ± 0.19	0.77 ± 0.18	0.83 ± 0.11	0.92 ± 0.06	1.17 ± 0.08**	1.26 ± 0.16	1.26 ± 0.09*	1.24 ± 0.16	1.27 ± 0.14

Values are expressed as mean ± SEM. *P<0.05 and **P<0.05, compared with control group.

Table S3. hTERT relative mRNA expression in RIRI-PA1-3 and RIRI-PA1-50 after AcMNPV-hTERT infection for 7 and 14 days.

Group	Control	7d MOI 1	7d MOI 5	7d MOI 10	14d MOI 1	14d MOI 5	14d MOI 10
**RIRI-PA1-3 cells**	-	1.06 ± 0.26	11.04 ± 2.67	17.36 ± 6.65*	10.51 ± 1.71	21.50 ± 1.40*	41.84 ± 7.56***
**RIRI-PA1-50 cells**	-	1.10 ± 0.32	0.72 ± 0.04	1.34 ± 0.09	0.48 ± 0.17	1.29 ± 0.21	0.39 ± 0.11*

Values are expressed as mean ± SEM. *P<0.05 and ***P<0.001, compared with 7d MOI 1 group.
